# Researcher-decision-maker partnerships in health services research: Practical challenges, guiding principles

**DOI:** 10.1186/1472-6963-12-280

**Published:** 2012-08-28

**Authors:** Anne Hofmeyer, Catherine Scott, Laura Lagendyk

**Affiliations:** 1 Higher Degrees by Research, School of Nursing & Midwifery, University of South Australia, GPO Box 2471, Adelaide, South Australia, 5001, Australia; 2 Departments of Community Health Sciences & Sociology, University of Calgary, 2500 University Dr. NW, Calgary, AB T2N 1N4, Canada; 3 Leading Practices & Innovation, Alberta Health Services, Calgary, Canada; 4 Senior Knowledge Management Consultant, Alberta Health Services, #400, 1509 Centre Street SW, Calgary, AB, T2G 2E6, Canada

**Keywords:** Researcher-decision-maker partnership, Collaborative research teams, Health services research, Ethics, Insider research

## Abstract

**Background:**

In health services research, there is a growing view that partnerships between researchers and decision-makers (i.e., collaborative research teams) will enhance the effective translation and use of research results into policy and practice. For this reason, there is an increasing expectation by health research funding agencies that health system managers, policy-makers, practitioners and clinicians will be members of funded research teams. While this view has merit to improve the uptake of research findings, the practical challenges of building and sustaining collaborative research teams with members from both inside and outside the research setting requires consideration. A small body of literature has discussed issues that may arise when conducting research in one’s own setting; however, there is a lack of clear guidance to deal with practical challenges that may arise in research teams that include team members who have links with the organization/community being studied (i.e., are “insiders”).

**Discussion:**

In this article, we discuss a researcher-decision-maker partnership that investigated practice in primary care networks in Alberta. Specifically, we report on processes to guide the role clarification of insider team members where research activities may pose potential risk to participants or the team members (e.g., access to raw data).

**Summary:**

These guiding principles could provide a useful discussion point for researchers and decision-makers engaged in health services research.

## Background

Funding organizations around the world increasingly require health services research proposals to include researcher-decision-maker partnerships; that is, to build a collaborative research team of individuals that represent relevant disciplines, decision-makers and stakeholders from industry, local communities (individuals and populations), and professional organizations [1-4]. Collaboration between researchers and decision-makers is one approach to generate research questions – and answers - that are relevant to the decision-makers’ real-life problems. It has been argued that involving the end users of research (such as decision-makers) in the entire process helps move the benefits of research evidence into action [2,4-6]. To achieve these potential benefits, it is imperative that we capture and share processes that effectively address challenges that emerge from collaborative research relationships.

In our experience, researcher-decision-maker partnerships facilitate health services research in multiple ways. For example, we find a collaborative approach to research promotes interest and participation in the research (both the project at hand, and research in general) by the decision-makers and their relevant stakeholder networks; and, the decision-maker partners provide the research team with a thorough understanding of the context under study, improving the quality and local relevance of the knowledge generated. A corollary of these benefits is the potential challenges – which we experienced in recent work. For example, by inclusion of decision-makers who were locally active in the cases under study, we were faced with dilemmas about access to the research data. Additional challenges were associated with gaining and sustaining participation of relevant decision-makers: a few decision-makers may be asked to participate in a number of separate but related research projects in their area of responsibility which may limit their ability to actively participate; and, during the time-frame of the study, decision-maker roles evolved and changed so that the area of study was no longer within their area of responsibility thus limiting their ability to meaningfully engage.

We found minimal guidance from funders and in the literature about how to clarify the role scope, participation and data access by various members of the partnership, especially those who have previous or current connections with the research setting and/or potential participants. A small body of literature has discussed issues that pertain to conducting research in quality, evaluation, and staff development in one’s own setting (i.e., “insider” research) but does not specifically address the potential pitfalls in collaborative research teams [7-11].

In this article, we highlight some of the challenges that arose for members who collaborated in a researcher-decision-maker partnership conducting health services research in Alberta, Canada. We first discuss the concepts of researcher-decision-maker partnerships. We then describe our program of research and the issues that arose. Notably we found scant literature to guide methodological and ethical issues in collaborative research teams, although the “insider” research literature provided some direction. Subsequently, we outline a range of practical ethical approaches to manage potential pitfalls and guide the participation of research team members who have insider connections with the research setting. These guiding principles aim to ensure the confidentiality and anonymity of participants’ data and provide clarity for team members. We encourage others to contribute to the needed discussion on the practical challenges that may arise when working in researcher-decision-maker partnerships.

## Discussion

### Researcher-decision-maker partnerships in health services research

Researcher-decision-maker partnerships are known to improve the use of research evidence in decision-making (policy, clinical, governance) and health research proposals with diverse team members are increasingly required by funders [1,3]. Many health services research teams now feature a mix of professions with varying types of decision-maker involvement by stakeholders representing clinical services, industry, local communities and professional organizations [1-3]. Following Lomas and Ross, we define “decision-makers” in our research as health system managers and policy-makers [2,3] and for this article use “researcher-decision-maker partnerships” and “collaborative research teams” interchangeably. The types of decision-maker involvement in the research can vary from “formal support” to being a “responsive audience” to that of an “integral partner” [3;p.S2:29].

In order to build diverse collaborative research teams that are primed to examine complex health problems, researcher team leads invite individuals to participate in different membership roles. This assumes teams include members that work inside and outside the study setting - organisation or community [9]. Members are chosen and their role is based on the individuals’ anticipated contributions, value to interpret meanings [9,11] and specific expertise in areas such as:

1. Sensitizing concepts [12] underpinning the research study (e.g., theoretical models, social context, policy; culture);

2. Methods and analytical approaches used in the study (e.g., mixed methods); and,

3. Organizational and/or practice (e.g., profession/discipline knowledge and expertise; position and influence in the organization and/or clinical practice area; established local and community knowledge, networks and linkages).

Given the criteria by which they are chosen it is plausible, and perhaps even desirable, that some team members could have previous or current links with the study organization/community.

## Researcher-decision-maker partnership in Alberta

### The example of CoMPaIR

CoMPaIR: *“Context and Models in Primary Healthcare and their Impact on Interprofessional Relationships*” is a program of research funded by the Canadian Health Services Research Foundation (REISS Grant ID – RC2 – 1474–09) with support also received from the former Calgary Heath Region (now part of Alberta Health Services), the former Alberta Heritage Foundation for Medical Research (now Alberta Innovates Health Solutions) and the University of Calgary. Ethical approval to conduct this study was received from the Conjoint Health Research Ethics Board, University of Calgary, Alberta, Canada (CHREB Ethics ID 20356).

The CoMPaIR collaborative research team is an example of a researcher-decision-maker partnership that was deliberately configured to include academic (i.e., university-based) members as well as decision-maker partners closely associated with primary healthcare (PHC). The researcher team lead (co-author Scott) was employed within the health system and held adjunct appointments at the local university. The dual roles facilitated a network of academic and decision-maker relationships from which the collaborative research team was built. The roles of the decision-makers were intended to: ensure the approach to research was appropriate for the context and the research questions were relevant; provide linkages with the PHC communities under study; contextualize the research findings; and, to facilitate dissemination and uptake of the research findings. It was anticipated that the researcher team lead, project manager, and collaborative research team members would contribute their expertise at various times throughout the study and in the translation of findings into suitable products for the various stakeholder groups. This means that some team members would be more active whereas others might have a more marginal or focused role to help achieve specific activities at key times in the research [13].

CoMPaIR was designed as a series of case studies of geographically based networks providing primary healthcare in Alberta, with each network considered as one case. Initial data collection included individual interviews with key stakeholders in the case. It quickly became apparent that numerous members of the CoMPaIR collaborative team were named and discussed in the case data; moreover, we found that some team members had links with the research setting that posed potential risk for interviewees or the team members. For example, some team members were participating, or had previously participated, in research projects other than CoMPaIR. Examples of the pre-established or dual relationships [7] included:

Team members or their work being mentioned by interviewees.

Team members who had existing employer and/or employee relationships with other team members or interviewees; and,

Work relationships that had previously or currently existed between team members and others who have influence in the case being studied.

In addition, during the timeframe of CoMPaIR, the roles and responsibilities of some team members changed and this altered their ability to participate actively in the program of research.

These challenges presented ethical issues related to ensuring the privacy, confidentiality and anonymity of participants’ data [14] and the integrity of the research process. For example, it is possible that participants would be unwilling to share openly about their experiences if they knew that a person such as a senior manager would have access to the raw unprocessed data and interview recordings. Even stripped of obvious identifiable information [14] it would be difficult to maintain confidentiality when interviewees disclosed detailed data about the case context. We had to manage this issue while ensuring the trustworthiness and credibility of the data was enhanced through interpretations of meanings by insider team members [9] who had subjective knowledge of the research setting [11]. In some instances, changes in team member roles meant that they were no longer supported to actively participate in the research as it was not relevant to their new role nor were they able to meaningfully contribute to understanding the evolving research context. This necessitated review of our originally proposed research processes and the roles of CoMPaIR team members, especially those who were decision-maker partners.

### Insider research

We consulted the literature for insights about how to understand and address the research processes and proposed roles of the collaborative research team members. A small body of literature discusses the merits and pitfalls of conducting research projects with participants with whom the researcher has some form of prior or existing knowledge and/or relationship [7-11]. This could be in terms of professional links with individuals such as teacher/student links or employer/employee relationships. In addition, the researcher could have some form of prior or existing links with the organization or site where the research is to be undertaken which may afford them insider knowledge about culture and organizational politics. The term ‘insider research’ refers to the time when a researcher “conducts studies with populations, communities, and identity groups of which they are also members" [10;p.439]. Potential pitfalls and approaches that researchers can employ to mitigate problems are explored in the literature. The focus is on issues that assume the researcher is the person who is directly involved in data collection and has access to the raw unprocessed data.

As discussed, research-decision-maker partnerships or collaborative research teams typically include members who have previous or current links with the research setting/organization/community. Often research assistants are employed to collect data such as individual and/or focus group interviews, as was the case in our research program in Alberta. One problem that arose for us was that some team members themselves were named and discussed in the qualitative data [8]. If disclosed, the nature of the discussions could potentially identify participants, posing an increased degree of risk for some team members or interviewees; or, could affect work or relationships in the setting in which decision-maker team members were actively involved. Additionally, we faced the issue of changes in team member roles influencing their ability to actively and meaningfully participate in the research. This latter issue required relatively straightforward discussions related to changes in team membership with some people withdrawing from the research team and new members joining. The former issue, however, (i.e., naming research team members as *participants in the case*) required a more considered approach and is the focus of this paper. Insider research literature focuses on addressing issues related to the researcher conducting the recruitment, data collection, analysis and reporting. We did not find guidelines in the research literature to manage this specific issue in the context of partnership research teams, so we developed and implemented guiding principles in our research.

### Guiding principles

We used ethical dialogue to guide the exchange between team members to plan good decision-making in the aforementioned circumstances [15]. Dialogue is described by Isaacs [16] as the art of thinking together to enhance shared meanings and foster new ways of seeing and common understandings of issues which could form a basis from which to think and act. Through ethical dialogue we created a definition for insider research team members who were *participants in the case*. Members of the collaborative research team were considered *participants in the case* when they were actively working in or closely associated with the case under study, or were named in the data. We then clarified the role scope, limitations and contribution by members of the collaborative research team who were considered *participants in the case*.

For example, team members who were *participants in the case* had restricted access to data. Agreement on this approach was achieved through open dialogue with team members about the issue and potential options for its resolution. An alternative that was rejected was that these team members withdraw their participation. As a result, we created an analytic team that was comprised of the researcher team lead, research manager and assistants; they were the only individuals who had access to the raw unprocessed data. This meant that no team members other than the members of the analytic team, had direct access to the data. The role of the analytic team was to ensure data was presented in a non-identifiable format for collaborative team members [17].

In this way, the confidentiality and anonymity of interviewees was assured so that they would have confidence to speak openly without fear of identification or retribution*.* Additionally, protection of members of the collaborative research team who had pre-established links with the research setting was paramount. The challenge was to provide the collaborative team members with sufficient information about the study data to allow them to contribute the expertise, knowledge and experience for which they were recruited to the team. To achieve this outcome, the process of ethical dialogue continued. Only data summaries about the research questions or topics of interest were provided to collaborative team members. Where specific data was considered to be of value for a broad audience and was clearly identifiable the content was shared only with the express consent of the relevant participants. If a team member wanted more information about a topic, those who had access to the raw data would provide a more in depth analysis or focused response, while still maintaining confidentiality and anonymity.

During data collection, some individuals were cautious during their interview about what information could be shared but few raised concerns related to sharing information with individuals on the research team. These concerns were addressed using the strategies described above. In addition, key stakeholders from each case were given an opportunity to review the case description included in the final report, and participated in a meeting with the PI and another member of the analytic team to review the case description and address any concerns.

Using this approach, we did not encounter issues related to differences in interpretation of the data (i.e., between those with access to the data and those without access). If such an issue had arisen, we proactively planned to: a) have those with access explore the data for more explanatory detail; and, b) when necessary, request that a neutral analyst review the data and provide their interpretations.

Given the dearth of literature on the practical challenges of working in collaborative research teams, we developed guiding principles that researchers and teams could adapt and adopt to supplement the ethical guidelines that govern the conduct of research projects in various countries and/or jurisdictions. These guiding principles aim to address potential pitfalls, ensure ethical data management, trustworthiness and credibility of the data, and the integrity of researcher-decision-maker partnerships.

### 1. When planning the study and writing the proposal

Identify individuals with pre-established links to the research setting (organization, community). This could include the principal investigator/researcher team lead. Identify other team members who could lead phases of the research that may have potential risk for the principal investigator /researcher team lead and/or the participants (i.e., where the team member is a *participant in the case*) (Personal communication: Cummings, September 3, 2008).

Develop clear expectations about the role scope, contribution, limitations and participation by team members considered *participants inthe case*[7].

As a collaborative team, and using the process of ethical dialogue, consider the implications of any limitations on the team members with links to the research setting. Will it still be possible to achieve the research study objectives?

Seek agreement that collaborative team members will engage in a process of self-reflection through dialogue and writing to acknowledge subjective bias, values, familiarity, agendas, taken-for-granted assumptions and perceptions about the contextual issues and cultural influences associated with the research setting [8,10,11].

### 2. When conducting the study

Include information about study purpose, role clarification, definition, scope and limitations of team members considered *participants in the case* in the recruitment letters for potential participants. The aim is to foster trust and reduce role confusion and misconceptions [8].

Participants must be assured that team members considered *participants in the case* will not have access to the original data or interview recordings and that anonymity and confidentiality will be upheld during the project and when reporting findings [8].

Distribute anonymized and de-identified data summaries to team members who are considered *participants in the case*.

Ask team members to continue to engage in self-reflection and ethical dialogue throughout the research project.

### 3. Contribute to the development of successful collaborative research teams

Assess and share methods of working within collaborative research teams where members such as decision-makers were *participants in the case*.

Foster ethical dialogue with groups and individuals who have a stake in building a body of knowledge to address real-life issues (i.e., funders, researchers, decision-makers, policy-makers, government agencies).

## Summary

This article contributes to the emergent body of knowledge about ethical issues that may arise in the case of partnerships between researchers and decision-makers that are designed to investigate real-life problems in health services. The value of including members of the research setting in the team has been affirmed in the knowledge translation and exchange literature and contributes to data credibility and implementation. While the number of collaborative research projects continues to increase globally, guidelines for working within teams that include decision-makers must also receive attention. This paper discussed the merits and potential pitfalls for collaborative research teams to consider when members of the team are insiders or *participants in the case*. We contribute to the emergent literature by proposing a set of guiding principles that researchers may adapt and adopt to manage the potential risk for study participants when members in researcher-decision-maker partnerships have pre-established relationships in the research setting.

## Competing interests

The author(s) declare that they have no competing interests.

## Authors’ contributions

CS conceived and designed the study and was the principal investigator for the study. She participated in the creation of the guiding principles and drafted the manuscript. AH co-created the guiding principles with CS, and helped to draft the manuscript. LL analyzed the qualitative data, reviewed and applied the guiding principles, and helped to draft the manuscript. All authors read and approved the final manuscript.

## Pre-publication history

The pre-publication history for this paper can be accessed here:

http://www.biomedcentral.com/1472-6963/12/280/prepub
